# Small-scale volcanic aerosols variability, processes and direct radiative impact at Mount Etna during the EPL-RADIO campaigns

**DOI:** 10.1038/s41598-020-71635-1

**Published:** 2020-09-16

**Authors:** Pasquale Sellitto, Giuseppe Salerno, Alessandro La Spina, Tommaso Caltabiano, Simona Scollo, Antonella Boselli, Giuseppe Leto, Ricardo Zanmar Sanchez, Suzanne Crumeyrolle, Benjamin Hanoune, Pierre Briole

**Affiliations:** 1 Laboratoire Interuniversitaire des Systèmes Atmosphériques-LISA, UMR CNRS 7583, Institut Pierre Simon Laplace, Université Paris-Est Créteil, Université de Paris, Créteil, France; 2 Istituto Nazionale di Geofisica e Vulcanologia, Osservatorio Etneo, Catania, Italy; 3 Consiglio Nazionale delle Ricerche, Istituto di Metodologie per l’Analisi Ambientale, Tito Scalo, Potenza Italy; 4 Istituto Nazionale di AstroFisica, INAF Osservatorio Astrofisico di Catania, Catania, Italy; 5 Université de Lille, UMR CNRS 8518-LOA-Laboratoire d’Optique Atmosphérique, Lille, France; 6 Université de Lille, UMR CNRS 8522-PC2A-Physico-Chimie des Processus de Combustion et de l’Atmosphère, Lille, France; 7 Laboratoire de Géologie, École Normale Supérieure, Paris, France

**Keywords:** Atmospheric optics, Volcanology, Environmental impact, Atmospheric chemistry, Geochemistry

## Abstract

The aerosol properties of Mount Etna’s passive degassing plume and its short-term processes and radiative impact were studied in detail during the EPL-RADIO campaigns (summer 2016–2017), using a synergistic combination of observations and radiative transfer modelling. Summit observations show extremely high particulate matter concentrations. Using portable photometers, the first mapping of small-scale (within $\sim 20\,\hbox {km}$ from the degassing craters) spatial variability of the average size and coarse-to-fine burden proportion of volcanic aerosols is obtained. A substantial variability of the plume properties is found at these spatial scales, revealing that processes (e.g. new particle formation and/or coarse aerosols sedimentation) are at play, which are not represented with current regional scale modelling and satellite observations. Statistically significant progressively smaller particles and decreasing coarse-to-fine particles burden proportion are found along plume dispersion. Vertical structures of typical passive degassing plumes are also obtained using observations from a fixed LiDAR station constrained with quasi-simultaneous photometric observations. These observations are used as input to radiative transfer calculations, to obtain the shortwave top of the atmosphere (TOA) and surface radiative effect of the plume. For a plume with an ultraviolet aerosol optical depth of 0.12–0.14, daily average radiative forcings of $-\;4.5$ and $-\;7.0\,\hbox {W/m}^2$, at TOA and surface, are found at a fixed location $\sim 7\,\hbox {km}$ downwind the degassing craters. This is the first available estimation in the literature of the local radiative impact of a passive degassing volcanic plume.

## Introduction

Volcanoes, through their varied spectrum of internal and macroscopic activity (from passive degassing to explosive eruptions), emit complex plumes of gaseous and particulate effluents. These emissions interact with the atmosphere in many ways. They have an important impact on the atmospheric composition^1^, the formation, life-cycle and properties of clouds^2^ and regional to global climate^3^. The main actors in the volcanogenic modulation of the Earth’s radiative balance and, then, climate are the long-lasting highly reflective sulphate aerosols (SA) formed from the gas-to-particle conversion involving volcanic sulfur dioxide ($\hbox {SO}_2$) emissions. The effect of volcanic SA produced by moderate-to-strong eruptions with plume injections in the stratosphere has been identified as one of the largest sources of uncertainty in our understanding of recent global climate change trends^4^. On the contrary, the regional impact on the air quality and climate of persistent degassing volcanoes, despite probably important in some cases, is not yet well understood or quantified.

One notable example of such potential *high-regional-impact volcanoes* is Mount Etna, Italy. Etna is one of the most important emitters of natural pollution on Earth, accounting for about 10% of the global average volcanic emissions of carbon dioxide and $\hbox {SO}_2$^5^. Its activity is characterised by continuous passive degassing and episodic explosive eruptions. The total mass of gaseous sulfur compounds emitted by Etna is estimated to be $0.7 \times 10^6$ tonnes of sulphur per year, which corresponds to about ten times the anthropogenic sulphur emissions in the same area^6^. Due to the considerable altitude of its five active craters (up to about 3,300 m), an important amount of its emissions are released in the free-troposphere, where the characteristic $\hbox {SO}_2$ lifetime and the related spatiotemporal scales of their impact are significantly larger than in the boundary layer^7^, where other pollutant sources, e.g. anthropogenic pollution, are active. It has been recently shown that the remote atmosphere in the Mediterranean, at distance of hundreds kilometers from Etna, brings the fingerprint of its passive and eruptive activity^8^. In particular, Etna’s emissions are found responsible of statistically significant larger $\hbox {SO}_2$ concentrations and smaller aerosol particles downwind with respect to background conditions^8^. Small and reflective aerosols can be readily associated to SA of volcanic origin^9^. This modulation of the aerosol layer properties is expected to systematically affect the radiative balance at the regional scale and then regional climate^10,11^.

Etna’s activity is particularly interesting because the Mediterranean basin is a climatic sensitive area^12^. The Mediterranean aerosol and radiative budget^13^ and hydrological cycle^14^ are presently the objects of intensive studies. Nevertheless, the possible important impact of Etna on the atmospheric composition and regional climate in the Mediterranean area is not yet systematically studied. The systematic quantification of this impact must necessarily pass through a deep understanding of the emission source and near-range processes (from the active craters to a few km distance). Near-source processes are responsible for the initial physico-chemical processing of the emissions and largely determine the volcanic aerosols properties impacting at larger spatial scales^15^. The volcanic aerosol properties can thus display large variabilities, along the plume dispersion, at relatively small spatial scales^16^. As such, this source and near-source characterisation, once clarified, can be used as input to further analyses of the chemical and micro-physical evolution of the emissions to secondary aerosols (including SA) and their impacts at the regional scale.

### The EPL-RADIO campaigns

The EC (European Commission)-funded EPL-RADIO project (Etna Plume Lab-Radioactive Aerosols and other source parameters for better atmospheric Dispersion and Impact estimatiOns) is dedicated to the source/near-source characterisation of Etna in terms of atmospheric aerosols. The project targets emission processes, from inner degassing mechanisms to aerosol near-source characterization^17^. The estimation of small-scale impacts is also a scope of EPL-RADIO. In the context of EPL-RADIO, three measurement campaigns have been carried out at Etna active craters and the surrounding area, during summers 2016 (campaigns C1 and C2) and 2017 (campaign C3). The experimental set-up for the three campaigns is summarized in Table 1. The volcanic aerosol source has been characterised by determining the size-resolved volcanic aerosol content with: (1) direct sampling with cascade impactors, (2) an optical particle counter (OPC), (3) two MicroTops-II (MII) sun photometers, one operating in the ultra-violet (UV)/near infrared (NIR) spectral ranges (MIIOM model) and one in the visible/NIR (MIISP model), (4) Fourier transform infrared (FTIR) spectroscopy, mainly to characterise the primary fraction of the emitted SA with respect to the secondary SA produced by in-plume conversion of $\hbox {SO}_2$ emissions, and (5) a multi-wavelength polarimetric scanning elastic/Raman LiDAR (Light Detection And Ranging) system at a fixed station, Serra La Nave observatory (SLN, $\sim$ 7 km from the active craters). The portability of MII has been exploited to provide short-term characterisation of the optical properties of the plume in an extended area around the active craters (from craters edge to $\sim$ 20 km downwind). Thanks to the scanning capabilities of the LiDAR system, three-dimensional aerosols information has been obtained from SLN station. To constrain formation/evolution processes of the aerosol in the plume, complementary gaseous composition and environmental conditions have been characterised using: (1) the mentioned FTIR and a UV spectrometer (UVS), that provided $\hbox {SO}_2$ column amounts and proportions of other volcanic effluents (ratios with respect to $\hbox {SO}_2$), (2) MII observations of water vapour (MIIOM and MIISP) and ozone (MIIOM). Emission fluxes of volcanic $\hbox {SO}_2$ have been constantly monitored, during the campaigns, by the FLAME (FLux Automatic Measurement) network of UV scanning spectrometers, operating on the flanks of Etna^18^.

**Table 1:** Experimental set-up for EPL-RADIO campaigns C1, C2 and C3.

Date	LiDAR	MII	Direct sampling	OPC	Gas observations
**Campaign C1**					
30/06/2016	Raman + Elastic		@BNC crater		UVS: @BNC crater
					FTIR: @BNC crater
01/07/2016	Raman + Elastic				
**Campaign C2**					
14/07/2016		1: distal			
18/07/2016		3: plume traverse			
19/07/2016	Elastic	2: transect	@BNC crater		UVS: @TDF
					FTIR: @BNC crater
20/07/2016		2: transect			UVS: @SLN
					FTIR: @SLN,TDF
22/07/2016			@V, NEC craters		
**Campaign C3**					
18/07/2017		2: distal, very distal			FTIR: distal (with MII)
19/07/2017	Raman + Elastic	1: distal			UVS: distal
					FTIR: distal (with MII)
20/07/2017		2: distal	@BN, VOC craters	@BN,VOC craters	UVS: @BN,VOC craters
					FTIR: @BN,VOC craters
21/07/2017		2: distal, proximal (lateral)			FTIR: distal (with MII)

The instruments and operations are described in the “Methods” section. For LiDAR and MII, operational modes are mentioned. For direct samplings, OPC and gas observations, explicit measurements locations are indicated: *BNC* Bocca Nuova, *NEC* Nord Est and *VOC* Voragine craters, *SLN* Serra La Nave, *TDF* Torre Del Filosofo. During C2 only a MIIOM has been operated; during C3 a MIIOM and a MIISP operated side-by-side.

The volcanic activity of Etna during the C1 and C2 campaigns (June and July 2016) was characterized by quiescent degassing following the effusive activity of May 2016. Only on 20/07/2016, discontinuous ash puffs were observed^19^. This quiescent degassing phase lasted until a volcanic event occurred on 7 August 2016, after the end of C2 campaign, when a new vent near the eastern rim of the VOC (Voragine crater) opened, producing high-temperature degassing. During the C3 campaign (July 2017), Etna showed minor and sporadic explosive activity with occasional ash emission at a vent opened on 15 December 2016, in the former saddle separating the SEC (South East crater) and NSEC (New South East crater) cone. The range of passive degassing and minor explosive activities covered by EPL-RADIO campaigns allows the investigation of very typical conditions of Mount Etna volcano.

## Results and discussion

### Summit observations: emissions

**Figure 1:**
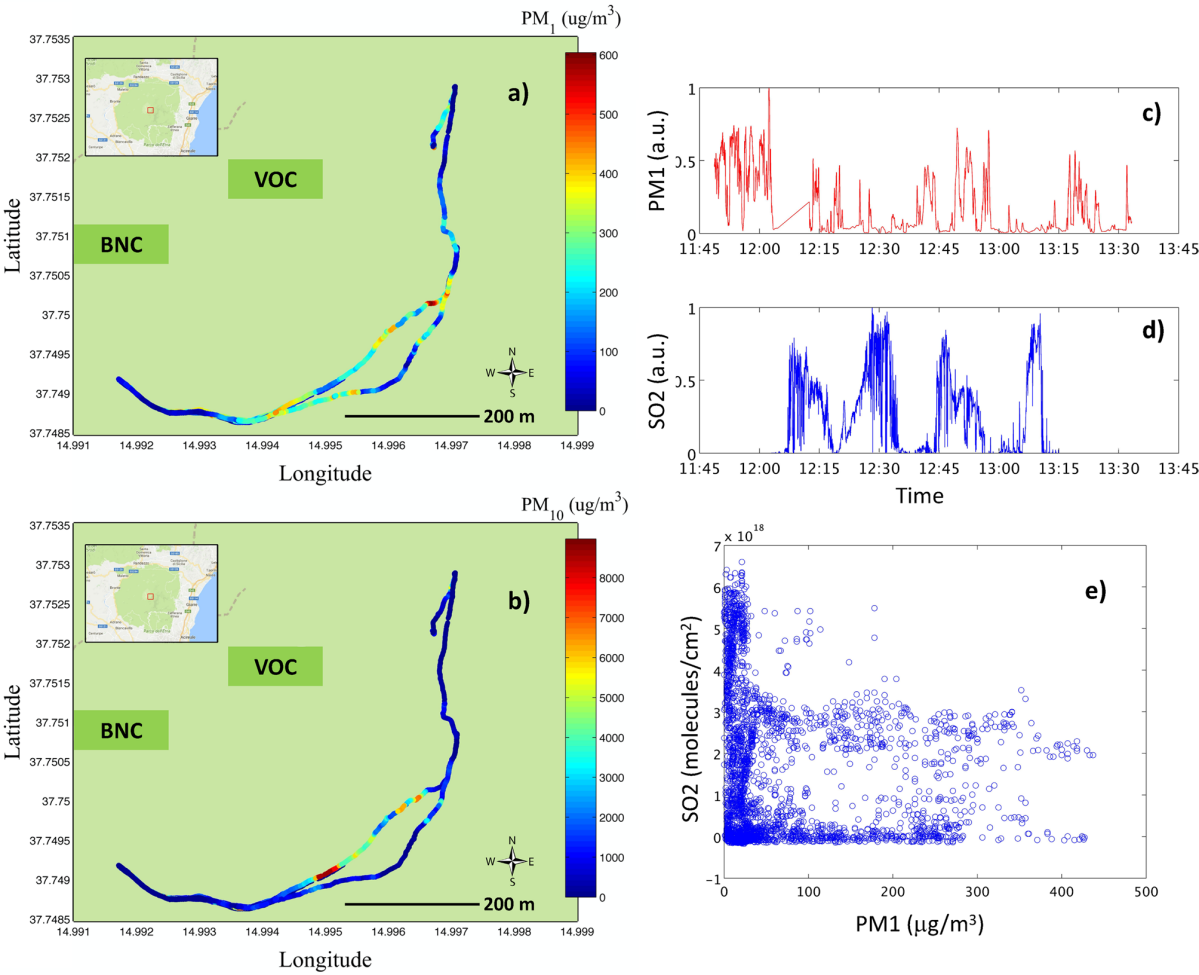
Fine ($\hbox {PM}_1$, **a**) and coarse ($\hbox {PM}_{10}$, **b**) particle matter concentration measured with OPC near the central BNC and VOC craters (approximate craters position indicated in the maps) during a double plume traverse on 20/07/2017. The background maps of panels a and b are generated with Google Maps (https://www.google.com/maps/, Map data $\copyright 2020$ Google). Fine particle matter concentration (**c**) and $\hbox {SO}_2$ time series (**d**) and scatter plot of simultaneous observations (**e**).

A first target of EPL-RADIO is the proximal aerosol plume characterisation, i.e. at Etna summit. Figure 1a,b shows $\hbox {PM}_1$ and $\hbox {PM}_{10}$ (particulate matter with diameter less than 1 and $10\,\upmu \hbox {m}$) concentrations measured with the OPC at Etna summit (BNC and VOC craters) on 20/07/2017. Etna’s plume was directed towards the south-east direction. Two plume traverses were realised on that particular day. The $\hbox {PM}_1$ concentrations were around $100\,\upmu \hbox {g/m}^3$ for most of the traverse and reached up to $600\,\upmu \hbox {g/m}^3$ in what can be identified as the approximate center of the summit plume. The $\hbox {PM}_{10}$ concentration reached up to 8,000$\,\upmu \hbox {g/m}^3$ in a limited-sized location. Results from chamber testing have shown that these low-cost sensors can underestimate $\hbox {PM}_1$ mass concentration and overestimate $\hbox {PM}_{10}$ at these extreme values (see “Methods” section). However, the observed concentrations are of the same order of magnitude as for previous measurements at Etna’s summit^20,21^. At similar passive degassing conditions, Allen et al.^20^ observed mass concentrations between $\sim \;$ 1,400 and $200\,\upmu \hbox {g/m}^3$, in the coarse mode (diameter $\hbox {D}_{p} > 3.5\,\upmu \hbox {m}$), and between $\sim 150$ and 4,500$\,\upmu \hbox {g/m}^3$, in the fine mode ($\hbox {D}_{p} < 3.5\,\upmu \hbox {m}$), at varying distances from the near-summit to $\sim \;10\,\hbox {km}$ distance. Similarly, very high concentrations have been observed for other volcanoes, like the Popocatepetl volcano (total suspended particles $\sim \;1,440\,\upmu \hbox {g/m}^3$)^22^ or the Soufrière Hills ($\hbox {PM}_{10} = 100$–$500\,\upmu \hbox {g/m}^3$)^23^. Our observations confirm (and exceed) extremely large PM values for Etna’s summit and for similar volcanoes. These extremely big PM sources (including the most dangerous small-sized particle matter) have been associated in the past to degraded air quality and connected pulmonary diseases of the neighbouring people^22–24^. These health risks are still to be systematically assessed for Etna area, including for the persistent passive degassing activity.

One can note that the $\hbox {PM}_{1}$ and $\hbox {PM}_{10}$ hot-spots are not localized exactly in the same plume section. Therefore, the sources of fine and coarse particles may be different. Moreover, the column integrated $\hbox {SO}_{2}$ concentrations were simultaneously measured along the same path; time series of $\hbox {PM}_{1}$ and $\hbox {SO}_{2}$ concentrations are shown in Fig. 1c,d. The $\hbox {SO}_2$ measurements show very limited correlations with both $\hbox {PM}_1$ and $\hbox {PM}_{10}$ concentrations (see a scatterplot of $\hbox {PM}_1$ and $\hbox {SO}_2$ concentrations in Fig. 1e). This evidence contrasts with previous evidences of a strong correlation of total particle column and $\hbox {SO}_2$ et Etna near-summit^15^. Even if during this measurement session the volcanic plume was mostly flattened to the ground due to the moderate wind at the summit, it has to be mentioned that part of the plume can be missed from the PM observations because it is located at higher altitudes. While the wind was not extreme, during the session, it is still possible that some of the coarser aerosol observations are moderately affected by surface particles re-mobilisation. Due to these limitations and the lack of complementary observations of atmospheric oxidants or ultra-fine particles, the precise origin of the observed aerosol is not possible. Nevertheless, these results do not show clear evidence of formation of secondary aerosols. Primary aerosol emissions were found dominating, with respect to secondary aerosol formation, at proximal locations, for other passive degassing plumes like for Masaya volcano in the past^25^.

### Distal and itinerant observations: small-scale variability

**Figure 2:**
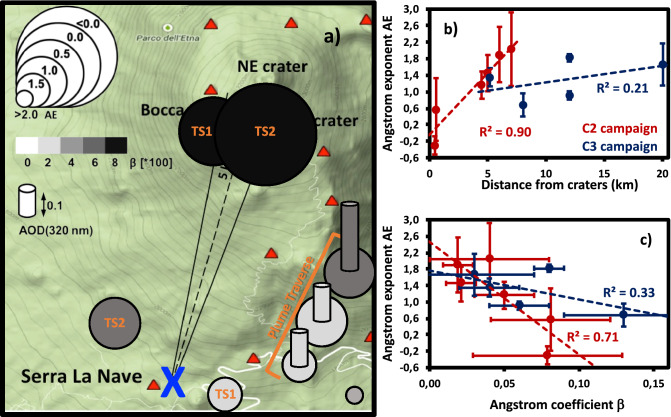
Summary of MII optical properties observations of Etna’s plume during C2 campaign (18–22/07/2016, **a**): AE (proportional to the size of the markers), $\beta$ (white–grey–black shade of the markers), UV AOD (height of the column during plume traverse). The different experiments are also individuated in (**a**): plume half-traverse and the two plume transects scans (TS1 and TS2). The location of the LiDAR station is indicated with a blue cross. The locations of FLAME stations are indicated with red triangles. The background map is generated with Google Maps (https://www.google.com/maps/, Map data $\copyright 2020$ Google). AE as a function of distance (**b**) and of $\beta$ coefficient (**c**), for MIIOM measurements collected during C2 campaign (in red) and for MIISP measurements collected during C3 campaign (blue). Individual points and error bars are associated to average values and variability for observations sessions at fixed locations (see Table 1). Regression lines are also reported (red and blue dashed lines) with their Pearson $\hbox {R}^2$ coefficients.

Getting new insights into the volcanic aerosol plume at *larger proximal* scale, i.e. under $\sim 20\,\hbox {km}$ from the degassing craters, is a further target of EPL-RADIO. Figure 2a shows a summary map of the MIIOM observations of the volcanic aerosols plume’s optical properties during C2 campaign. Aerosol optical depths (AOD) for Etna’s plume were derived at 320.5 nm (hereafter referred to as UV AOD) and 1,020.0 nm (hereafter referred to as NIR AOD), with the method described by Sellitto et al.^19^. Using UV and NIR AODs, the Ångström exponent (AE) and the Ångström $\beta$ coefficient are subsequently derived^19^. In general, shorter-wavelength AODs carry more information on the presence of smaller particles than longer-wavelength AODs^26^. The spectral variability of the AOD (and then of the extinction of radiation due to the presence of the aerosol) can be represented using the empirical Ångström law, as a function of AE and $\beta$:

1 $$\begin{aligned} \mathrm {AOD}(\lambda )=\beta \lambda ^{-\mathrm {AE}} \end{aligned}$$

Starting from the fundamental theory of particles-radiation interaction (Mie theory^26^), AE and $\beta$ can be considered as optical proxies for particles mean size and burden/composition, in the aerosol layer. Bigger AEs are linked to smaller particles, on average, in the sampled plume. While this relationship of AE and average particle size is generally true, precise studies on the average particle size evolution by means of AE observations may be complicated by the possible evolution of the overall particle size distribution, and in particular of the number of its modes. Log-normal size distributions with two^20^ or three modes^15^ have been found at near-crater locations for passive degassing volcanoes. On the contrary, mono-modal size distributions, in the accumulation mode (typical mean particles size around 0.1–$0.5\,\upmu \hbox {m}$), have been found at distal locations^27^ . The $\beta$ parameter is representative of the AOD value at wavelength of $1.0\,\upmu \hbox {m}$. For the particle types in volcanic plumes and the typical size distributions discussed above, $\beta$ is representative of the coarse particles (mainly ash) in the plume. Thus, while its interpretation is more complex than for the AE, in our present study $\beta$ can be linked to the burden of the coarse aerosol in the layer, bigger $\beta$s being representative of bigger burdens. Despite the intrinsic complexity and uncertainty in their interpretation, the two Ångström parameters are widely recognised as diagnostic tools for volcanic plumes morphological and microphysical properties and their inherent evolution processes^28^. The average values of AE and $\beta$ are estimated for each measurement session at each location, during C2, as listed in Table 2 and are shown on the map. Thus, Fig. 2a represents the small-scale variability and evolution with distance of the average size and coarse aerosol burden of a volcanic plume. During C2 campaign a clear image of progressively more dominant smaller particles, associated with a smaller burden of coarse particles, is apparent from crater to increasing distances. At the same time, the UV AOD has a different behavior during both campaigns C2 and C3, with slightly increasing values in the distal field, thus indicating a possible slight increase of the finer aerosol content in the plume. This result is consistent with previous airborne remote observations of Spinetti and Buongiorno^16^, that have shown a similar increasing trend, for increasing distance from the crater, of the AE and a near-constant to slightly increasing trend of the distal (starting from a few km downwind the craters) short-wavelength AOD (at about 400 nm), during passive degassing activity of Mount Etna volcano. A substantial variability of the plume properties is found at spatial scales that are smaller than typical grid-points of chemistry/transport models and pixels of satellite observations. This reveals that processes are at play which are not represented with current regional scale modelling and observations. To get further insights into the small-scale aerosol variability, in Fig. 2b,c we show the MIIOM AE (C2 campaign) and MIISP AE (C3 campaign), as a function of the distance from craters and of simultaneous measurements of $\beta$. The error bars in Fig. 2b,c represent the standard deviation of the average AE and $\beta$, thus representing their variability for each measurement session/location. Results shown in Fig. 2b,c confirm the picture drawn before for C2 campaign: progressively smaller particles and decreasing coarse aerosol burden are found along plume dispersion in the first $\sim$10 km around Etna’s degassing craters. The correlation of the occurrence of smaller particles with increasing distance and with thinner plumes is significant (R$^2$ correlation coefficients 0.90 and 0.71, respectively). This can be attributed to relatively quick sedimentation of coarser ash particles (bigger than a few micrometers) emitted by discontinuous ash puffs (observed during C3 campaign and on 20/07/2016 during C2 campaign) and, possibly, by sustained gas-to-particle conversion of $\hbox {SO}_2$ volcanic emissions to secondary SA and subsequent grow by condensation (up to a few hundreds nanometers). These two processes are expected to concur to the progressive modification of the size distribution towards smaller average sizes. During C2 campaign, volcanic activity was based on a prevalent passive degassing and $\hbox {SO}_2$ emission rates were significant yet very variable (average values of 2,240.5 ± 882.5 t/day, observed by the FLAME network). Nevertheless, the same picture is not found for C3 campaign. Even if smaller particles sizes and coarse aerosol burdens are found with increasing distance from the emitting craters, the regression lines have less pronounced slopes and much smaller correlations (0.21 and 0.33) than for C2 campaign. This may be linked to the larger amount of emitted coarse ash particles and/or a smaller signal of gas-to-particles conversion to SA. For C3 campaign, sporadic explosive activity is found, with significantly larger ash emissions and with smaller $\hbox {SO}_2$ emission rates (average values of 1,315.9 ± 254.2 t/day), which can be limiting factors to secondary SA in-plume production. A similar negative correlation between the volcanic aerosol amount and average size, stronger for ash-free that ash-bearing plumes, as shown in Fig. 2c, was found during past field campaigns at different volcanoes, including Mount Etna^28,29^, and attributed to ash sedimentation and in-plume SA formation.

**Figure 3:**
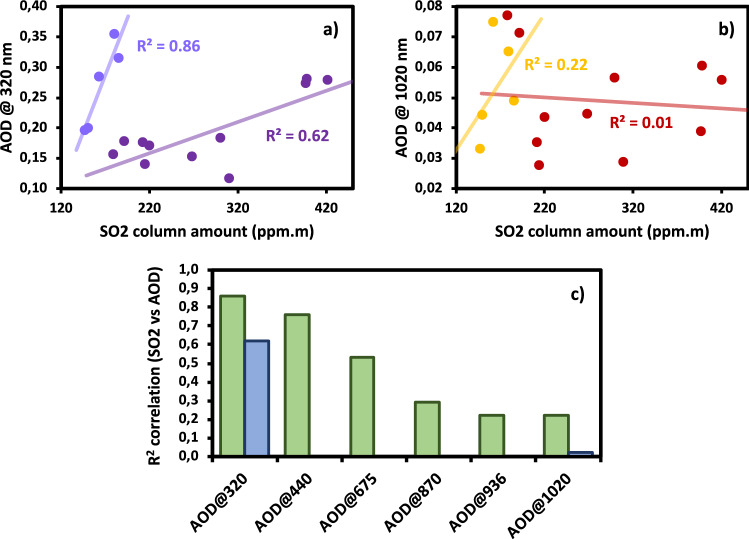
Scatter plot of MIIOM UV (**a**) and NIR (**b**) AODs and UVS $\hbox {SO}_2$ simultaneous observations, for C2 [indigo (**a**), and yellow (**b**)] and C3 campaign [mauve (**a**), and orange (**b**)]. Regression lines and respective Pearson correlation coefficients R$^2$ are also provided for each campaign and wavelength. Pearson correlation coefficients R$^2$ for correlations of simultaneous and co-located UVS $\hbox {SO}_2$ with respect to MIIOM/MIISP AODs observations at different wavelengths (**c**), for C2 (blue bars) and C3 (green bars).

Simultaneous side-by-side measurements of MIIOM and MIISP volcanic AODs, and UVS $\hbox {SO}_2$ column amounts, were carried out during both C2 and C3 campaigns, and are compared in Fig. 3. The UV AOD correlates much better than the NIR AOD to UVS $\hbox {SO}_2$ estimations (Fig. 3a,b). In general, the correlation decreases with wavelength and is only significant in the UV-to-shorter-visible range (Fig. 3c). As shorter-wavelengths AOD values are more strongly correlated with the presence of small-sized particles in the aerosol layer than longer-wavelengths AOD, and $\hbox {SO}_2$ is the main precursor of the tiny secondary SA, this is a strong indication of $\hbox {SO}_2$-to-SA conversion processes at play. In addition, UV and NIR AODs correlate both better to $\hbox {SO}_2$ during C2 than C3 campaign. This gives a further indication that possible $\hbox {SO}_2$-to-SA conversion is more marked during volcanic passive degassing conditions, with stronger $\hbox {SO}_2$ emissions, than in mildly explosive conditions, with weaker $\hbox {SO}_2$ emissions. New SA particle formation has been recently identified for Etna’s degassing plume, at larger spatial scales, by Sahyoun et al.^30^. It is important to mention that the presence of in-plume and background atmospheric aerosols in the line-of-sight of the UVS spectrometer might have an impact leading to either over- or under-estimating $\hbox {SO}_2$ column amounts measurements, by multiple scattering and effective path lenght reduction due to light dilution, respectively^31^. These effects are expected to be negligible for the observations carried out in this study. Multiple scattering by in-plume aerosols is expected to be important for aerosol extinctions significantly larger than those observed in our study. The light dilution effect depends on the background atmosphere and is not related to the plume AOD. In addition, for vertical observation geometry, this effect is largely spectrally independent. As we took care to carry out observations at as much as possible vertical geometry, this has a negligible effect on the results discussed above.

Besides a general short-term depiction of the geographical near-source variability of volcanic AOD for Etna, in Fig. 2a different experiments are identified: two plume transects (TS1 and TS2, see details in Table 2), i.e. rapid longitudinal plume scans, and a plume half-traverse, i.e. a rapid perpendicular plume scan from the plume centre to one periphery. For the plume half-traverse, additional indications of the measured UV AODs are given in the map. The dramatic change in average size and coarse aerosol burden of the plume during the transects (details in Table 2), in such a short time and small distance, can be readily attributed to the sedimentation of possible coarser ash particles and the rapid formation of secondary SA. Coarser particles are found during TS2 than TS1, due to the visible ash puffs during this measurement session. Some residual relatively-coarse ash particles might be present at distal TS2 location due to these more pronounced ash emissions, which are unlikely present during TS1-distal observation ($\hbox {AE} = 1.90\pm 0.67$, typical of extremely small particles). The larger relative uncertainties at proximal ($\sim 95\%$, TS1, and $\sim 60\%$, TS2) than distal UV AOD ($\sim 45\%$, TS1, and $\sim 35\%$, TS2), see Table 2, point at more inhomogeneous plumes near the sources. This plumes tend to get more and more temporally homogeneous with increasing distance, as different particles types (like fine ash) are removed from the plume along dispersion. Larger variabilities of volcanic plumes’ optical properties have been associated with *puffiness* of the plume and to the presence of ash^29^. The short-term atmospheric processes *smooth* the plume, in terms of its properties. The plume half-traverse revealed the perpendicular structure of Etna’s plume, during a typical passive degassing situation. The burden is maximum at the plume core (UV AOD $\sim \;0.2$) and steadily decreases towards the plume periphery (UV AOD $\sim \;0.1$, i.e. half the burden than at the plume core). Correspondingly, the particle mean size decreases from the core to the periphery. New SA particles formation is expected to be more effective at the plume periphery, i.e. in presence of lower concentrations of pre-existing aerosols that can act as condensational sink^30^.

**Table 2:** Details about TS1 and TS2 MIIOM transect scans during C2 campaign. Date, approximate time, observation session location, UV AOD, AE are reported.

Date	Approximate time	Site	UV AOD	AE
19/07/2016	10:30	0.5 km SE of SLN (distal)	$0.138\pm 0.064$	$1.90\pm 0.67$
19/07/2016	11:30	TDF (proximal)	$0.213\pm 0.207$	$0.57\pm 0.76$
20/07/2016	09:30	TDF (proximal)	$0.060\pm 0.036$	$-0.30\pm 0.22$
20/07/2016	11:00	1 km NW of SLN (distal)	$0.161\pm 0.054$	$1.16\pm 0.33$

### Distal and itinerant observations: vertical distribution

**Figure 4:**
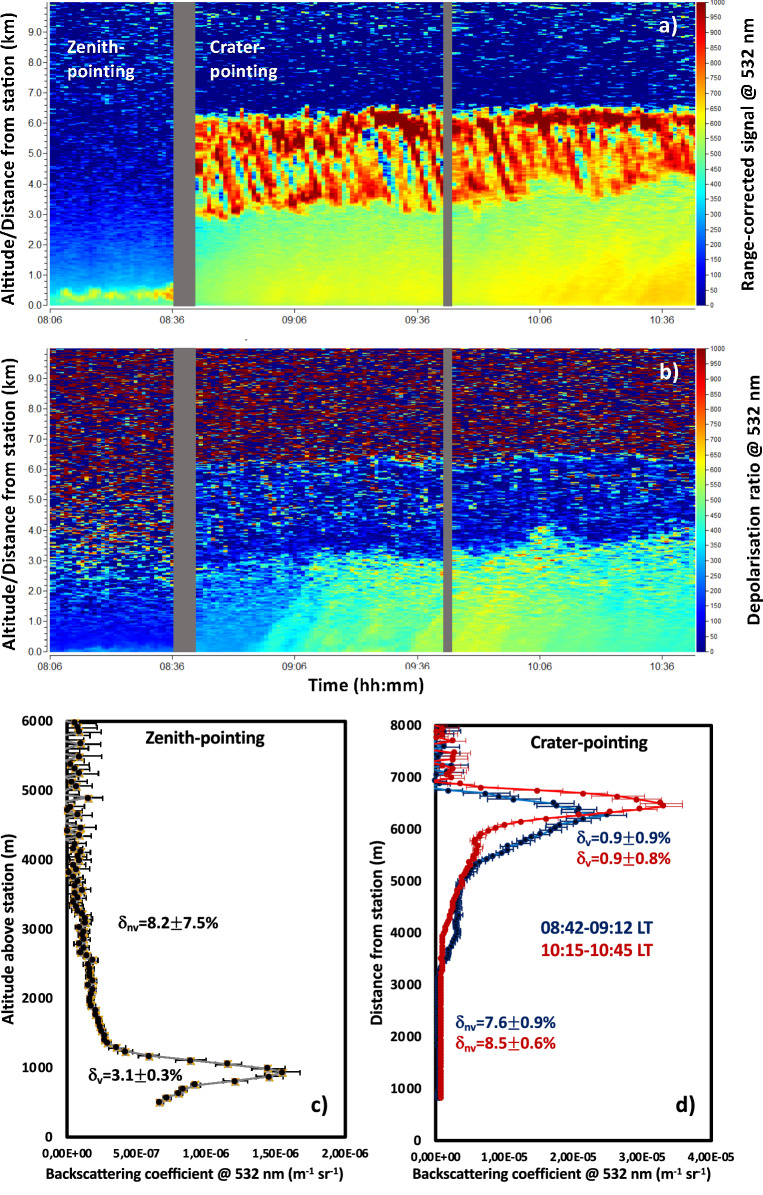
Time series of LiDAR observations from SLN station (blue cross in Fig. 2, 19/07/2016) of range-corrected signal (**a**) and depolarisation ratio (**b**) at 532 nm, for zenith-pointing (as a function of the altitude) and crater-pointing (as a function of the distance from station) geometries. For both geometries, the average backscattering coefficient vertical profile at 532 nm is also shown (**c**, **d**), along with the average depolarisation ratio for volcanic-($\delta _{\mathrm {v}}$) and non-volcanic-affected ($\delta _{\mathrm {nv}}$) identified vertical ranges. For crater-pointing observations, two average profiles and mean depolarisations are shown for different time intervals (08:42–09:12 local time, in blue, and 10:15–10:45 local time, in red).

The vertical distributions of volcanic aerosols is further studied using LiDAR observations from the fixed SLN station. Figure 4 shows typical aerosol vertical structures at passive degassing conditions (19/07/2016). Back-trajectories analyses (not shown here) show that this profile is mostly affected by local air masses, at all altitudes. No larger scale features, like desert dust transport events, are observed at all altitude ranges. Zenith- and crater-pointing observations allow a detailed three-dimensional characterisation of the passive degassing plume. These observations reveal an aerosol layer at low altitude over the station (from near-ground to $\sim$ 2 km, aerosol signal peaking at $\sim$ 1 km, zenith-pointing observations), possibly covering the whole line of sight, from station to crater (crater-pointing observations, aerosol signal over the whole line-of-sight and peaking at $\sim$ 5–7 km, i.e. at and near the crater itself). The core of the volcanic degassing plume is characterised by weakly depolarising aerosols ($\sim 3$ and 1%, for zenith- and crater-pointing observations, respectively), thus indicating the predominant presence of spherical droplets, e.g. SA. The weak signal-to-noise ratio after plume crossing, i.e. visible in the average crater-pointing observations, may point at partially absorbing particles near the crater area, possibly SA embedding a more absorbing fine ash core.

On 19/07/2016, quasi-simultaneous MIIOM and LiDAR zenith observations have been realised at SLN station. This allows the direct comparison of the AODs obtained with the two instruments. The MIIOM UV AOD (at 320.5 nm) is $0.14\pm 0.06$, for the volcanic plume. Using a LiDAR ratio (LR) of 48 sr, as done previously, for volcanic plumes, at the same station^32^, we obtain a LiDAR VIS AOD (at 532 nm) of 0.06. This value, scaled using the quasi-simultaneous AE estimations made with MIIOM at SLN, produces a full-column LiDAR UV AOD (at 320.5 nm) of 0.16. While a limited impact of other local aerosol sources at the very lowest altitudes cannot be excluded, we attribute to the volcanic plume the aerosols observed up to 2 km over SLN station and obtain a LiDAR UV AOD of 0.12, for the volcanic plume. The consistency of quasi-simultaneous MIIOM photometric and LiDAR UV AODs, for the volcanic plume, validates our choice of LR and the LiDAR observations themselves.

### Radiative transfer modelling: direct impact on the regional climate

**Figure 5:**
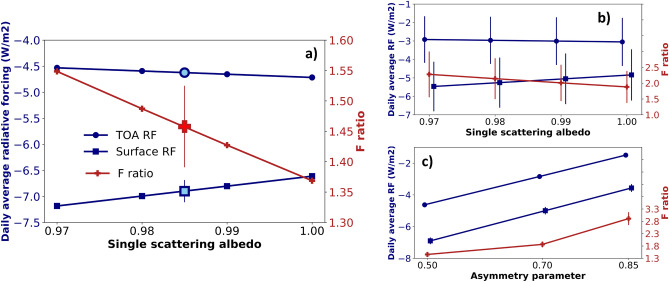
Equinox-equivalent daily average TOA (blue dots) and surface (blue squares) radiative forcing, and their f ratio (red crosses), as a function of the single scattering albedo assumptions, for asymmetry parameter $\hbox {g}=0.50$. Their mean values (by averaging all estimations with the different single scattering albedos, for $\hbox {g}=0.50$) is also shown with 1-standard-deviation error bars (filled blue dot, blue square and red cross) (**a**). Equinox-equivalent daily average TOA and surface radiative forcing and f ratio, averaged over all values of g and as a function of SSA (**b**), and averaged over all values of SSA and as a function of g (**c**).

The near-source impact of the volcanic aerosol plume on the shortwave radiative balance is then estimated using the UVSPEC radiative transfer model. The measured average volcanic aerosols extinction profile of the AMPLE LiDAR at SLN station (19/07/2016), and validated by co-located photometric observations, is used as a representative near-source plume at typical passive volcanic degassing conditions. Even though here we use precise vertically-resolved estimations of the aerosol extinction of Etna’s plume, hypotheses are necessary for other optical properties not accessible from the synergy of LiDAR and photometer measurements. The absorption and scattering properties of the aerosol layer is based on the hypothesis of a predominance of tiny, highly-reflective SA^10^. The single scattering albedo (SSA), an optical proxy of the aerosol absorption, is set at typical values for high-reflective sulphate aerosols. Values around 1.00 have been reported for these particles in the shortwave spectral range^33^. As the uncertainty on this assumption is relatively high—the plume can contain a fraction of more absorbing ash particles, even if this is unlikely (see previous discussion on LiDAR and MIIOM observations, and volcanic activity type)—four groups of simulations are performed using SSA ranging from 0.97 to 1.00, with 0.01 SSA increments. The angular distribution of the plume-scattered radiation, can be modeled by scattering moments based on Heyney-Greenstein functions, corresponding to a given asymmetry parameter g (the intensity-weighted average of the cosine of the scattering angle, that can be obtained with the Mie theory^34^). In the shortwave spectral range, considering the expected mean size of the plume’s particles, a typical value of the asymmetry parameter, for very weakly-absorbing particles like freshly nucleated/condensed SA, is 0.50^35^. This value has been used as a reference for our simulations. Nevertheless, the uncertainty on this assumption can be large and so simulations for two additional values of g, 0.70 and 0.85, are performed. Based on these considerations, 12 simulations are performed, with four values of SSA and three values of g. It must be noted that the spectral variability of both SSA and g are not taken into account in this study. For all simulations, the surface reflectivity, which is an important parameter determining the local shortwave radiative balance, is set at 0.1 (wavelength-independent), which is a typical value of vegetated surfaces, like in the volcano near-surroundings. Figure 5a shows the clear-sky equinox-equivalent daily average top of the atmosphere (TOA) and surface radiative forcing and their ratio (called *f ratio*) as a function of the SSA, and their SSA-averaged values, for a 0.50 asymmetry parameter g. The TOA radiative forcing of the passive degassing plume is near-independent on the absorption properties of the volcanic aerosols, and has an average value of $\sim \;-4.5$ W/m$^2$, thus indicating a consistent cooling of the local climate system. The surface radiative forcing of the passive degassing plume is more dependent on the absorption properties of the volcanic aerosols, and has an average value of $\sim \;-7$ W/m$^2$. Correspondingly, the f ratio depends quite strongly on the absorption properties of the plume and has an average value of $\sim$ 1.45. The average TOA and surface radiative forcing and f ratio, as a function of the SSA, for simulations with the three values of g, are shown in Fig 5b. The same radiative forcing parameters, as a function of g, for simulations with the four values SSA, are shown in Fig 5c. From the comparison of these two latter panels, it can be seen that the parameter that brings the largest uncertainty is the asymmetry parameter g. A larger cooling effect is found for smaller values of g, which means smaller particles and then larger scattering back to space. For larger particles, values of f ratio up to near 3.0 are found, which means a consistent energy stuck into the atmosphere, that can lead to local heating of plume-occupied air masses. The effect of SSA is less strong but a clear trend of larger values of the surface radiative forcing and f ratio for smaller SSA, which means more absorbing particles, is found. The TOA radiative forcing is near-independent on the SSA. For more absorbing particles, values of f ratio up to about 2.0 are found.

These estimations of the radiative forcing can be compared with more distal estimations of the radiative forcing for Etna’s emissions during explosive eruptions. The radiative forcing efficiency (radiative forcing per AOD unit) has been estimated, at TOA and surface: (1) for a SA-dominated plume, at $\sim 300\,\hbox {km}$ downwind Etna for a moderate eruption (25/10/2013), at $\sim$ 40–50 and 50–$70\,\hbox {W}/(\hbox {m}^2\hbox {AOD})$^11^, and (2) for an ash-containing plume, at $\sim 400\,\hbox {km}$ downwind Etna for a relatively strong eruption (03/12/2015), at $\sim \;112$ and $145\,\hbox {W}/(\hbox {m}^2\hbox {AOD}$)^36^. These values, scaled at the AOD observed in this case, lead to the forcings, at TOA and surface, of $\sim \;-5$–6 and $-6$–$8\,\hbox {W/m}^2$, for the SA-dominated, and $\sim$ $-13$ and $-17\,\hbox {W/m}^2$, for the ash-dominated plume. Our estimations, for a proximal passive degassing plume, are more in the magnitude range of purely SA plumes. The proximal radiative forcing efficiency of Mount Etna’s passive degassing plume is comparable to the very distal (some hundreds km) efficiency of explosive SA-dominated plumes, though with a factor ten smaller AOD^37^.

## Methods

### Volcanic gas characterisation using a FTIR spectrometer and a UV Spectrometer

The gas composition of the plume, in terms of the $\hbox {SO}_2/\hbox {HCl}$ ratio, was determined using an Open-Path Fourier Transform Infrared (OP FTIR) spectrometer, using the sun as IR radiation source. The FTIR spectra were collected using a M4400 spectrometer of the Midac Corporation with ZnSe beam splitter, with $0.5\,\hbox {cm}^{-1}$ resolution and an operational spectral range between 500 and 5,000$\,\hbox {cm}^{-1}$. Solar occultation^38^, in which the plume is between the sun and spectrometer, provides information on the plume composition in terms of IR active species and their ratios. This applies notably to $\hbox {SO}_2$ and HCl, two gas species with negligible concentrations in the free atmosphere but, conversely, abundant in volcanic emissions. The collected spectra were evaluated using a non-linear least-squares fitting algorithm build upon a two-layer (volcanic plume and atmospheric background) forward radiative transfer model^39^. The total uncertainties on the retrieved concentrations is estimated at values $<4\%$. The average molar ratios, generally calculated using at least 100 individual spectra, are determined by scatter plots of retrieved column amounts of $\hbox {SO}_2$ against HCl. The daylight bulk $\hbox {SO}_2$ emission rates from the summit craters of Etna was measured by the FLAME-Etna network (FLux Automatic Measurements)^40^. The network consists of ten UV scanning spectrometer stations installed at an altitude of $\sim \;900$ m a.s.l. on the flanks of Etna at a mean distance of $\sim 14\,\hbox {km}$ from the summit craters. Open-Path UV (OPUV) spectra are reduced on site applying the DOAS (Differential Optical Absorption Spectroscopy) technique and using a modelled clear-sky spectrum^18^. From inverted data, $\hbox {SO}_2$ emission rates are derived. Uncertainty in rates ranges between $-22$ and $+36$%. OPUV spectra were also collected from itinerating positions, following the plume dispersion, with a fixed field of view on selected days. The UV spectrometer (UVS) was mounted on a tripod with the telescope orientated $20^{\circ }$ from zenith, to achieve closest line of sight with the sun photometer. The total integration time for the collection of these spectra ranged from 2 to 30 s. Column amounts of $\hbox {SO}_2$ were retrieved applying the standard DOAS method.

### Particulate matter concentration in-situ measurements using an OPC

The portable OPC device used to measure particle matter concentrations at Etna summit, during C3 campaign, was assembled at the Laboratoire d’Optique Atmosphérique, Lille. It is built around a commercial HK-A5 laser $\hbox {PM}_{2.5}/\hbox {PM}_{10}$ sensor, which counts all the particles with an optical diameter between 0.3 and $10\,\upmu \hbox {m}$ and classifies them into six size bins. The measurement frequency is 1 Hz. The device communicates to a cell phone via Bluetooth with a specifically developed application. The application provides the geo-positioning, stores the results for further analyses, and allows real time visualization of the measurements. The accuracy of the measurements has been checked regularly since early 2017 using laboratory-grade instruments (GRIMM 1.108). The accuracy is highly dependent on the aerosol concentration within the measurement cell. Due to coincidence errors, $\hbox {PM}_1/\hbox {PM}_{10}$ measurements are expected to be underestimated/overestimated, respectively, in polluted environments. $\hbox {PM}_1$ correlation coefficients are $\sim 1.0$ for $\hbox {PM}_1$ concentrations $<100\,\upmu \hbox {g/m}^3$ and fall $\sim \;0.7$ for higher $\hbox {PM}_1$ concentrations. $\hbox {PM}_{10}$ correlation coefficients $\sim \;1.0$ for $\hbox {PM}_{10}$ concentrations $<20\,\upmu \hbox {g/m}^3$ and fall $\sim 1.7$ for higher $\hbox {PM}_{10}$ concentrations. These tests have been performed with laboratory generated particles that can be strongly different from real atmospheric aerosols. As the corrections may change in specific environments, like within the Etna’s plume, in this work we used the OPC output with no correction, and we take them as indicative.

### Aerosol optical properties and water vapour observations using MII sun photometers

Aerosol optical properties at Etna were derived using two hand-held MicroTops-II (MII) sun photometers^41^ operating at different spectral regions. During C2, a MIIOM (MicroTops-II Ozone Monitor) was used, while during C3 a MIISP (MicroTops-II Spectro Photometer) operated side-by-side with the MIIOM. Both instruments measure direct sun radiance at relatively small field of view ($2.5^{\circ }$). The MIIOM operates primarily in the UV spectral range, in three channels at $305.5\pm 0.3$, $312.5\pm 0.3$ and $320.5\pm 0.3$, with nominal full width at half maximum (FWHM) of $2.4\pm 0.4\,\hbox {nm}$, to derive ozone vertical columns. The AOD at 320.5 nm is derived using the methodology described by Sellitto et al.^19^. Two additional channels in the near infrared (NIR) ($936.0\pm 1.5$ and $1,020.0\pm 1.5\,\hbox {nm}$, both with nominal FWHM of $10.0\pm 1.5\,\hbox {nm}$) are also present in MIIOM. These two channels are used to derive water vapor vertical content and NIR AOD, respectively. From UV and NIR AODs, the Ångström exponent AE and Ångström $\beta$ coefficient are derived^19^. The theoretical uncertainties of UV AOD, AE and $\beta$ are estimated at values $<\pm 0.035$ ($<\pm 12\%$), $<\pm 0.2$ ($<\pm 15\%$) and $<\pm 0.015$ ($<\pm 15\%$), for typical conditions during the campaigns^19^. The MIISP operated during C3 has three bands in the atmospheric-window visible spectral range ($440.0\pm 1.5$, $675.0\pm 1.5$ and $870.0\pm 1.5$), used to derive AODs at these wavelengths, as well as the same two NIR channels as for MIIOM. All MIISP channels have nominal FWHM of $10.0\pm 1.5\,\hbox {nm}$. The AE and $\beta$ discussed in this paper are derived using the channels at 440 and 870 nm. The theoretical uncertainties of visible AOD from MIISP are $<\pm 0.02$^42^, while the theoretical uncertainties of MIISP derived AE and $\beta$ are expected to be comparable or smaller to those obtained with MIIOM. The instruments operated during C2 and C3 campaigns have been pre-calibrated with a Langley method at Mauna Loa Observatory, Hawaii, (MIIOM: July 2014, MIISP: October 2011). MIIs are very light (about 600 g) thus allowing prompt changes of locations during the campaign, necessary for the different plume-tracking and plume-scanning measurement protocols described in the Results section.

### Aerosols observations using the AMPLE LiDAR system at Serra La Nave station

LiDAR measurements were carried out using the innovative AMPLE (Aerosol Multi-wavelengths Polarization LiDAR Experiment) scanning system, aimed to study volcanic plumes from Etna^32^. The AMPLE system is part of EARLINET (European Aerosol Research Lidar Network) and is devoted to special measurement campaigns mainly during eruptions^43^. The AMPLE system is based on a Nd:YAG diode-pumped laser source whose fundamental wavelength is frequency doubled and tripled. The laser beams, with average optical power of 0.6 W at 355 nm, 1.5 W at 532 nm, and 1.0 W at 1,064 nm, are beamed simultaneously into the atmosphere with 1 kHz rate. The receiver system is based on a telescope in a Dall–Kirkham configuration whose primary elliptic mirror has a diameter of 250 mm. The system has been designed to collect elastic LiDAR returns at 355 and 532 nm and the N$_2$ Raman echoes at 386 and 607nm. The LiDAR is also able to acquire simultaneously the depolarization signal at two different wavelengths (355 and 532 nm). During the EPL-RADIO campaigns, the LiDAR system was operated at SLN “M.G. Fracastoro” site (14.97 E, 37.69 N, 1,735 m a.s.l.), about 7 km away from Etna’s summit craters. Several LiDAR observations at the zenith and towards the volcanic plume were performed with 1 min time integration. Data were analyzed with time integration of 30 minutes in terms of aerosol backscattering profiles ($\beta _{\mathrm {L}}$) at 355 nm and 532 nm, and extinction profiles ($\alpha _{\mathrm {L}}$) at 355 nm. The $\beta _{\mathrm {L}}$ coefficient was retrieved from nighttime observations by means of the Raman method^44^, based on simultaneous detection of both elastic and N$_2$ Raman LiDAR echoes. From daytime measurements the $\beta _{\mathrm {L}}$ coefficient was obtained applying the Klett–Fernald algorithm^45^. The retrieval of the $\alpha _{\mathrm {L}}$ coefficient was performed using the N$_2$ Raman LiDAR signal measured in nighttime conditions^46^. Furthermore, simultaneous aerosol backscatter and extinction data at 355 nm was used to estimate the LiDAR ratio (LR), a key parameter for the aerosol type classification. The analysis of the components of the backscattered radiation at 532 and 355 nm polarized along the direction perpendicular and parallel to the laser beam polarization allowed the derivation of the aerosol depolarization vertical profiles ($\delta _{\mathrm {L}}$)^47^.

### Radiative effect calculations using the LibRadTran suite

The shortwave surface and top of the atmosphere (TOA) direct radiative effect is estimated using the UVSPEC radiative transfer model and the LibRadtran package^48^, available at the following website: http://www.libradtran.org/doku.php. The radiative transfer equation is solved with the SDISORT method, the pseudo-spherical approximation of the discrete ordinate method (DISORT)^49^. Surface and TOA direct and diffuse shortwave spectra are computed in the range 300.0–3,000.0 nm (0.1 nm spectral resolution). We use the input solar flux spectra of Kurucz^50^. The atmospheric state in terms of the vertical profiles of temperature, pressure, humidity and gas concentration is set as for the AFGL (Air Force Geophysics Laboratory) climatological standard summer mid-latitude atmosphere^51^. Molecular absorption is parameterised with the LOWTRAN band model^52^, as adopted from the SBDART code^53^. We performed clear-sky simulations. We performed a baseline simulation, with this setup and a background atmosphere without volcanic aerosols. Then we add the measured volcanic aerosols extinction coefficient profiles of the AMPLE LiDAR. For baseline and volcanic plume configurations, we simulate the radiative transfer at different solar zenith angles (SZA). The equinox-equivalent daily average shortwave TOA radiative forcing for the volcanic aerosol layer is calculated as the SZA-averaged upward diffuse irradiance for a baseline simulation without the investigated aerosols minus that with aerosols, integrated over the whole spectral range. The shortwave surface radiative forcing is calculated as the SZA-averaged downward global (direct plus diffuse) irradiance with aerosols minus baseline, integrated over the whole spectral range. Equinox-equivalent daily averages, i.e. based on same nighttime and daytime duration, are specifically calculated and discussed in this paper.

## Data Availability

All datasets generated during and/or analysed during the current study are available from the corresponding author on reasonable request.
